# Evidence of Phenotypic and Genetic Relationships between Sociality, Emotional Reactivity and Production Traits in Japanese Quail

**DOI:** 10.1371/journal.pone.0082157

**Published:** 2013-12-04

**Authors:** Julien Recoquillay, Christine Leterrier, Ludovic Calandreau, Aline Bertin, Frédérique Pitel, David Gourichon, Alain Vignal, Catherine Beaumont, Elisabeth Le Bihan-Duval, Cécile Arnould

**Affiliations:** 1 INRA, UR83 Recherches Avicoles, Nouzilly, France; 2 INRA, UMR85 Physiologie de la Reproduction et des Comportements, Nouzilly, France; 3 CNRS, UMR7247, Nouzilly, France; 4 Université François Rabelais de Tours, Tours, France; 5 IFCE, Nouzilly, France; 6 INRA-ENVT, UMR444 Génétique Cellulaire, Castanet-Tolosan, France; 7 UE1295 Pôle d’Expérimentation Avicole de Tours, Nouzilly, France; Institut Pluridisciplinaire Hubert Curien, France

## Abstract

The social behavior of animals, which is partially controlled by genetics, is one of the factors involved in their adaptation to large breeding groups. To understand better the relationships between different social behaviors, fear behaviors and production traits, we analyzed the phenotypic and genetic correlations of these traits in Japanese quail by a second generation crossing of two lines divergently selected for their social reinstatement behavior. Analyses of results for 900 individuals showed that the phenotypic correlations between behavioral traits were low with the exception of significant correlations between sexual behavior and aggressive pecks both at phenotypic (0.51) and genetic (0.90) levels. Significant positive genetic correlations were observed between emotional reactivity toward a novel object and sexual (0.89) or aggressive (0.63) behaviors. The other genetic correlations were observed mainly between behavioral and production traits. Thus, the level of emotional reactivity, estimated by the duration of tonic immobility, was positively correlated with weight at 17 and 65 days of age (0.76 and 0.79, respectively) and with delayed egg laying onset (0.74). In contrast, a higher level of social reinstatement behavior was associated with an earlier egg laying onset (-0.71). In addition, a strong sexual motivation was correlated with an earlier laying onset (-0.68) and a higher number of eggs laid (0.82). A low level of emotional reactivity toward a novel object and also a higher aggressive behavior were genetically correlated with a higher number of eggs laid (0.61 and 0.58, respectively). These results bring new insights into the complex determinism of social and emotional reactivity behaviors in birds and their relationships with production traits. Furthermore, they highlight the need to combine animal welfare and production traits in selection programs by taking into account traits of sociability and emotional reactivity.

## Introduction

Behavioral characteristics such as group structure, sexual behavior or response to humans have facilitated the domestication of animals and their adaptation to farming conditions [1]. However, domestication and thereafter evolution of farming practices have led to profound changes in the social environment of these animals. For example, despite being difficult to study in their native habitat, it appears that the Red Junglefowl (*Gallus gallus*), the ancestor of domestic hens, shows social structures with a small number of individuals of different ages and both sexes, with a strict hierarchy around a dominant male [2,3]. A similar social structure appears in feral fowl [4]. However, in modern farming systems, birds are mostly housed in large groups, sometimes of the same sex, and most often of the same age. These conditions may favor the expression of deleterious behaviors such as aggression, feather pecking and cannibalism in the most serious cases, which can affect both bird welfare and productivity [5,6].

The selection of domestic animals on production traits indirectly induces changes in behavioral traits [7]. Studying the genetic basis of these behavioral traits is an important step in understanding the propensity of each individual to express certain behavioral patterns and can lead to improvements in the animals’ housing environment or in the adaptability of animals to husbandry conditions. For this purpose, some experimental lines have been divergently selected for their propensity to express feather pecking in chickens [8], for their social reinstatement behavior or their duration of tonic immobility after a human physical constraint in Japanese quail [9]. Tonic immobility is considered as a measure of the level of fear [10,11]. Today, the techniques for detecting Quantitative Trait Loci (QTLs) allow progress to be made in understanding the relationship between behavior and genetics through identifying chromosomal regions of interest involved in the expression of behavioral traits. Thus, QTLs have been identified for the duration of tonic immobility in Japanese quail [12-14], or fearful behavior and social motivation exhibited in an unknown environment (open-field test) in chickens [15]. Genes involved in feather pecking behavior have also been identified [16,17]. Furthermore, domestication-related genetic effects on behavior have also been investigated through an intercross between Red Junglefowl and White Leghorn [18,19].

To our knowledge, no genetic studies to date have been conducted on the effect of introducing the criterion of social motivation in the selection process in birds. Nevertheless, this trait could influence other behaviors which need to be controlled under farming conditions such as sexual or aggressive behavior, and also emotional reactivity, which may all affect both bird welfare and production levels. The aim of our study on Japanese quail was to investigate the phenotypic and genetic relationships between several social behaviors (social reinstatement, response to social isolation, sexual motivation, aggression), behaviors measuring the emotional reactivity of the birds (reaction to an unknown object or to a human, tonic immobility reaction), their general level of activity and production traits (body weight and egg production). The interest of this study is that it combines all these measures on the same birds. The analyses took advantage of an F2 cross between two lines of quail divergently selected for their social reinstatement behavior [9] and showing differences in their social motivation under various conditions [20-23] and also in their emotional responses in some testing situations, such as response to a novel object [22]. Such an F2 cross shows a great variability in social behaviors due to the initial differences between lines, which facilitates the study of links between traits. Furthermore, considering an F2 cross rather than divergent lines alleviates the risk of observing links due to random occurrence, as a consequence for example of genetic drift, and allows the consequences of physical vicinity of genes to be investigated.

## Materials and Methods

### Ethics statement

All animal care and experimental procedures reported in this paper were in accordance with French and European regulations concerning animal experimentation, including authorization no. 37–129 from the French Ministry of Agriculture. The Experimental Unit where birds were kept is registered by the ministry of Agriculture with license number B-37-175-1 for animal experimentation. All behavioral tests were approved by the ethics committee in Animal Experimentation of Val de Loire (permit number 2011-07-10). This ethics committee is registered by the National Committee under the number 19.

### Animals

Two divergent lines bred and reared at the INRA experimental unit 1295 (UE PEAT, F-37380 Nouzilly, France) were used in the experiment. These lines with either high or low social reinstatement behavior (HSR or LSR, respectively) have been divergently selected on their propensity to rejoin a group of conspecifics when 10 days old [9] while maintaining a constant duration of tonic immobility across generations. They differ consistently on their social motivation under various experimental conditions and also on several aspects of their social behavior such as sexual motivation or aggressive behavior (for review see 23-25) and also notably on the characteristics of the social bond they develop [26,27]. A reciprocal cross of these two lines was done from the 49^th^ generation, using four HSR males and four LSR females to produce the HSR x LSR (H/L) cross and four LSR males and four HSR females to produce the LSR x HSR (L/H) cross. From this F1 generation, three H/L males were each mated to two H/L females and two L/H females, and three L/H males were mated to two H/L females and two L/H females. A total of 912 F2 quail chicks (452 males and 460 females) were produced across six batches separated by a 4-week interval. On average each female produced 40 chicks and each male produced 160 chicks. During the experiment two females from the F1 generation died and were replaced by individuals with a similar pedigree for the F2 production leading to 26 F1 females. In addition, nineteen HSR (12 males, 7 females) and twenty LSR (12 males, 8 females) quail were tested simultaneously with a batch of F2 quail in order to evaluate the differences between lines in the same context as for the F2 cross. 

### Housing conditions

F2 quail chicks were reared in groups of about 40 birds in battery cages until three weeks of age. At this age the birds’ sex was determined from plumage color. The quail were then individually housed in battery cages from three weeks old to the end of the experiment. Males and females were in the same room from three to five weeks old and thereafter they were housed in two completely independent rooms. Lighting was continuous for the first three weeks and temperature was progressively reduced from 38 to 20°C. In individual battery cages the birds were exposed to a light cycle of 12L:12D until five weeks of age and 16L:8D thereafter. Temperature was held constant at 20°C. Water and food were provided ad libitum. Feed was adapted to the physiological state of the bird (from day 0 to 21: commercial quail starter diet, STARGIB G111 first age, SFNA, 49160 Longué Jumelles, France; from day 21 to 35: growing diet; from day 35 to 65 for males or 210 for females: breeding diet; growing and breeding diets were both produced at the INRA experimental unit 1295, UE PEAT). The 19 HSR and 20 LSR quail were reared under the same conditions as F2 birds, except between 21 and 35 days of age when they were housed in same-sex pairs instead of individually.

### Testing procedure

Eight behavioral tests were performed on each quail from day 1 to day 62 of age. Quail were always tested individually. Tests used for the divergent selection of the HSR and LSR quail were conducted, i.e. the test of social reinstatement behavior, and the tonic immobility test [9]. As HSR seem to be more sensitive or reactive than LSR birds in some reactivity tests such as the novel object test [22], other tests measuring different aspects of sociality or emotional reactivity were also performed: firstly, response to social isolation, aggressive behavior toward conspecifics and sexual motivation, and secondly, response to a novel object and response to a human. In addition, we measured the birds’ general activity. Although there is no difference between the two lines for their general activity when observed under non-test conditions [27], the selection criterion used involved a distance run on a treadmill and this might have affected the general activity of the birds in addition to their social reinstatement behavior. 

#### Reaction to social isolation (day 1 to 3)

Quail were removed from their brooder cages and transferred in groups of 20 individuals to a room (A) adjacent to the testing room (B). In room A, they were housed in wooden cages measuring 63 cm x 38 cm x 28 cm (depth × width × height) with a wire mesh cover and wood shavings on the floor and containing two feed troughs and a drinker. Feed and water were provided ad libitum. Temperature was regulated using incandescent light-bulbs suspended above the cages. Temperature was maintained at approximately 37°C under the bulb. Quail were familiarized with these cages for at least one hour before testing. They were then tested in a wooden arena (51.5 cm x 42.5 cm x 30 cm) similar to the cage described above but without wire mesh cover and heated at the same temperature. A drinker (ø: 15 cm) and a feed trough (ø: 7 cm) similar to those used in room A were in the center of the arena with a free space between both (about 12 cm). The quail was gently placed between these two equipements, facing one length of the arena. After testing, quail returns in room A. Before, but also after testing, birds were always housed in group. When all the 20 quail have been tested, they returned to their brooder cage. Quail behavior was recorded using a camera suspended above the arena and connected to a computer equipped with the Ethovision tracking system (v XT7.0, Noldus Technology, Wageningen, The Netherlands). The total distance travelled (locomotor activity), the distance travelled in the periphery of the arena (6 cm along the walls) and the number of jumps along the walls were recorded for three minutes. The distance travelled in the periphery, referred to as DistIso in this study, was used as an indicator of the time spent pacing, which has been shown to be a relevant measure of social motivation by Schweitzer et al.[27].

#### Social reinstatement behavior (day 6 to 8) and tonic immobility (day 9 to 10)

The procedures used for these tests were similar to those used for the selection and described in detail in Mills and Faure [9]. Birds were transferred and housed in groups to room A, under the same conditions as for the social isolation tests and were then tested in room B. Social reinstatement behavior was assessed by measuring over a 5-minute period the distance (arbitrary unit) an isolated chick ran on a treadmill apparatus to rejoin a group of five conspecifics (see 9 for details). This variable is referred to as DistSR in this study. Duration of tonic immobility is a behavioral and physiological response modulated by frightening situations and is considered as a measure of the level of fearfulness [10,11]. Chicks were placed on their back in a U-shaped cradle and restrained for 10 s and the duration of tonic immobility was recorded. If a bird failed to right itself after 5 min a maximum score of 300 s was recorded. If tonic immobility was not induced after five attempts, a score of 0 s was recorded (see 9 for details). This variable is referred to as TI in this study. 

#### General activity inside rearing cages (day 23 to 30)

From day 23 to the end of the experiment, quail were individually housed. The behavior of each quail in its cage was measured using the scan sampling method between 23 and 30 days of age. Four times a day (9.00-10.00, 11.30-12.30, 14.00-15.00 and 16.30-17.30) two scans per bird were performed at an interval of 15 min. This procedure was repeated at 23, 27 and 30 days of age giving a total of 24 scans per bird. The observer sat 3 meters back from the front of the cages and noted if the quail was standing or lying to assess the level of general activity. The number of scans in which birds of two adjacent cages were interacting (lying in close contact along the wire mesh wall, eating simultaneously, or pecking one of its neighbors) was very low and was thus not analyzed. The variable used in the analyses was the percentage of time birds spent standing in the scans, referred to as STAND in this study.

#### Reaction to a novel object (day 37 to 38) and reaction to a human (day 42 to 43)

First, the usual feeder used for a series of eight cages was removed. Then, an individual feeder free of food and containing a novel object was put in front of each of the eight cages successively. The object was a 10 cm multi-coloured cylinder (ø: 2 cm) fixed vertically in the feeder (see 22 for details on the object). The feeder was designed so that the object could only be seen by one quail at a time, i.e. the two lateral sides have the same height than the cage. Furthermore, from day 35, lateral sides of the cages were opaque. The observer, positioned one meter from the cage inserted the feeder containing the novel object in front of the first cage and then recorded quail behavior for 2 min using the scan sampling method. Every 10 s the observer noted if the quail had touched the object, had passed its head through the wire of the front of the cage, without touching the object, or stayed inside the cage. At the end of the test, the feeder containing the novel object was removed and the same procedure was used for the next cage until the eight quail were tested. Then the usual feeder was put in place again and a new series of eight quail was tested. For phenotypic and genetic analyses, the number of scans in which the quail had its head through the wire of the front of the cage touching the object or not was used. This variable is referred to as HeadNO in this study.

The same procedure and observer as for the reaction to a novel object were used to investigate the reaction to a human, except that in this case the observer put his hand in the individual feeder in the same place as the object had been. The measures recorded were the same as for the novel object test.

#### Aggressive behavior (day 55 to 56)

To avoid injury and possible consequences of encounters with a conspecific on the following tests (dominance when winning, subordination if defeat), the measure of aggressive behavior was performed using a mirror. This procedure has already been used in other studies to assess social interactions [12,28]. Quail were tested in a rectangular arena (51.5 x 42 x 26 cm) made of PVC, protected by a curtain around it and lit with a 25 W incandescent light bulb. A mirror (41 x 24.5 cm) was sticked along one of its widths. The quail was gently placed in the center of the half side of the arena opposite to the mirror and facing the mirror. The quail was allowed to interact with its reflection for 2 min. The quail’s behavior was recorded using a digital camcorder suspended above the arena. Every 10 s we recorded whether the quail had pecked vigorously at the mirror (aggressive pecks) or had pecked gently (less vigorous pecks, with non-aggressive posture) [29]. Vigorous pecks were successive fast pecks sometimes associated with wing flapping and/or running or fast steps when approaching the mirror. This is intensive agonistic behavior [29]. Threats (stiff body posture with feathers lifted) and retreats were recorded but, due to their low frequency, were not used in the analyses. Male and female quail were tested for aggressive behavior. However, only data from males were used in analyses as expression of aggressive behavior in females was very low. The number of aggressive or gentle pecks is referred to as AgrP or GentleP, respectively, in this study.

#### Sexual motivation of the males (day 62)

This test was performed in the same arena and following the same procedure as for the aggressive behavior test, except that the mirror was removed and a stuffed female quail in a receptive posture was placed in the center of one of the half side of the arena. The tested quail was gently placed in the center of the other half side of the arena facing the lateral side of the stuffed female. The number of mounts over the 2-min period of the test was recorded. This variable is referred to as Mount in this study.

#### Production traits

Birds were weighed at 17 days of age (W17), just before being transferred to individual cages. They were also weighed after the last behavioral test, i.e. at 65 days of age (W65). Egg production was recorded daily for all the females until 30 weeks of age. The age when the first egg was laid (AFEgg) and the number of eggs laid (NEgg) until week 24 were recorded and the mean egg weight (WEgg) was calculated from eggs laid during weeks 12 and 13. 

### Statistical analyses

Normal distribution of the data was assessed by calculating the kurtosis and skewness parameters and by looking at the normal probability plot. As the distribution of several behavioral traits differed greatly from normality, non-parametric statistics were used. Phenotypic correlations between traits measured in the different tests were estimated by the Spearman Rank correlation using R software. The behavior of the HSR and LSR lines was compared using a Mann–Whitney U test with SYSTAT 8.0 (SPSS Inc., Chicago, IL, U.S.A.). Body weights of HSR and LSR lines were compared using t-tests as the data distribution was normal. 

The methodology to estimate genetic parameters usually implies a normal distribution of traits which was the case for DistIso and the different production traits. The duration of tonic immobility and the distance travelled on the treadmill were close to normality after log transformation and these transformed data were used for the analyses. As no effective transformation was obtained for the other behavioral traits, they were converted into classes before analyses with an appropriate threshold model [30]. The classes were constructed as follows: for the time spent standing, four classes of an equal number of individuals; for the other traits (HeadNO, Mount, AgrP, GentleP), three classes with a specific class corresponding to the “0” value and two classes of an equal number of individuals for values strictly superior to 0. 

The heritability (h^2^) and genetic correlations (r_g_) for behavioral and production traits were estimated for each combination of two traits using TM software [31] which can process continuous and categorical traits. Variance and covariance components (as well as the corresponding h^2^ and r_g_ parameters) were estimated using Gibbs sampling which consisted of a chain of 100,000 iterations, discarding the first 20,000 iterations and saving a sample every 20 iterations. The h^2^ and r_g_ estimates, as well as their standard errors, corresponded to the average and standard deviation of the parameters obtained from the 4,000 remaining iterations. For each iteration, the genetic parameters were estimated using the following linear mixed model: 

$$y_{ijkl}=\mu +b_{i}+s_{j}+m_{k}+a_{l}+e_{ijkl}$$

where y_ijkl_ is the observation for animal l, μ the overall mean, b_i_ the fixed effect of hatch i (i=1 to 6), s_j_ the fixed effect of sex j (j=1,2), m_k_ the maternal permanent environmental effect (k = 1 to 26) applied to body weight at 17 and 65 days, age of first egg and egg weight, a_l_ the additive genetic effect of the animal l (l = 1 to 944), and e_ijkl_ the residual term for animal l.

## Results

### Comparison of HSR and LSR lines

As shown in Table 1, HSR and LSR quail responses were highly divergent on most of the variables. The distance travelled on the treadmill was considerably higher in HSR than in LSR quail. This was also the case for the distance travelled in the periphery of the arena during the social isolation test. At the same time, HSR quail jumped significantly more than LSR birds. Regarding the tests on emotional reactivity, the duration of tonic immobility was significantly higher in HSR than in LSR quail. The number of scans in which the head of the quail passed through the wire of the front of the cage during the novel object test and in which quail had physical contact with the object was significantly lower in HSR than in LSR quail. This divergence between lines was not observed for the reaction toward a human test, whatever the variable. This test was not used in F2 analyses due to the low variability of the data (high level of 0 values). The number of aggressive pecks was higher in HSR than in LSR males, although the difference was not statistically significant. This absence of signification might be due to the low statistical power of the test linked to the low number of males tested (n=12 in each group). No significant difference was observed for gentle pecks which were performed only by two males out of 12 HSR birds and one male out of 12 LSR birds. Finally, no significant difference was revealed between the two lines for sexual motivation estimated by the number of mounts. HSR quail were significantly lighter (75.2 g ± 9.4 g, mean ± SD) than LSR birds (99.3g ± 8.1 g) at 17 days (p < 0.001) of age and also at 65 days of age (HSR : 193.5 g ± 26.6 g; LSR: 238.8 g ± 22.5 g ; p < 0.001). 

**Table 1:** Medians and interquartile ranges (Q1-Q3) for the behavior recorded in HSR and LSR quail.

	HSR			LSR				
	Q1	Median	Q3	Q1	Median	Q3	U	P**
**Social reinstament behavior:**								
Distance travelled (arbitray unit, DistSR)	810.5	1,033.0	1,262.5	18.5	45.5	78.5	362	**<0.001**
**Reaction to social isolation:**								
Distance travelled in periphery (cm, DistIso)	2,051.4	2,532.6	3,066.2	95.7	494.9	856.3	131	**<0.001**
Number of jumps	1.0	1.0	3.0	0.0	0.0	0.0	111	**0.003**
**Tonic immobility:**								
Duration (s, TI)	22.5	25.0	38.5	15.0	18.0	22.0	316.5	**<0.001**
**Reaction to a novel object:**								
Head passed through the wire of the front of the cage (no. of scans, HeadNO)*	0.0	0.0	0.0	0.0	1.5	8.0	92	**0.001**
Object touched (no. of scans)	0.0	0.0	0.0	0.0	0.0	3.0	111	**0.004**
**Reaction to a human:**								
Head passed through the wire of the front of the cage (no. of scans)*	0.0	0.0	2.5	0.0	0.0	4.0	164	0.404
Hand touched (no. of scans)	0.0	0.0	0.0	0.0	0.0	0.0	161.5	0.167
**Sexual motivation (in males):**								
Number of mounts (Mount)	0.5	8.0	13.0	0.0	3.5	12.0	83.5	0.501
**Aggressive behavior (in males):**								
Aggressive pecking (no. of scans, AgrP)	0.0	1.0	8.0	0.0	0.0	3.5	89.5	0.280
Gentle pecking (no of scans, GentleP)	0.0	0.0	0.0	0.0	0.0	0.0	78	0.514

N_HSR_ = 19 (12 males, 7 females) and n_LSR_ = 20 (12 males, 8 females) except for the test of social isolation where n_HSR_ = 11 (7 males, 4 females) and n_LSR_ = 12 (7 males, 5 females).

* Out of 12 scans, **: Mann-Whitney U test

### Distribution of the traits and phenotypic correlations in the F2 population

Distributions of behavioral and production traits are presented in Table 2 and Figure S1. The mean value for DistSR (487 arbitrary unit) was intermediate to those observed in the 52^th^ generation in the HSR (1459 arbitrary unit) and LSR (53 arbitrary unit) lines. In the case of tonic immobility duration (TI), F2 birds showed a lower mean duration (32 s) than those measured in the HSR and LSR lines from the 52^th^ generation (52 s and 55 s, respectively). 

**Table 2:** Descriptive statistics.

	DistSR	DistIso	TI	HeadNO	Mount	AgrP	GentleP	STAND	W17	W65	AFEgg	NEgg	WEgg
Number of birds	912	899	912	899	412	437	437	834	910	886	453	434	439
Minimum	0	5.4	0	0	0	0	0	45.8	74.0	157.0	36.0	60.0	9.7
Maximum	2,587	3,961	300	12	25	12	11	100	131.0	341.0	79.0	174.0	16.3
Mean	487	1,196	32.1	1.7	7.3	5.0	1.2	82.7	99.8	224.6	56.0	144.0	12.5
Standard deviation	509	722	23.7	1.9	6.8	5.0	2.1	11.2	8.5	30.1	6.0	18.0	1.1
Quartile 1	90	641	18	0	0	0	0	75	94.0	203.0	52.0	139.0	11.7
Median	310	1,096	26	0	7	4	0	83.3	100.0	221.5	55.0	150.0	12.5
Quartile 3	731	1,665	39	3	13	10	2	91.7	106.0	245.0	58.0	155.0	13.4
Skewness	1.32	0.65	3.81	1.98	0.40	0.29	1,99	-0.68	0.15	0.55	0.86	-2.00	0.10
Kurtosis	1.22	0.18	27.57	3.92	-1.06	-1.62	3.55	0.01	0.3	0,03	2.53	4.72	-0.20

DistSR: Distance travelled on the treadmill in the social reinstatement behavior test; DistIso: Distance travelled in periphery in the social isolation test; TI: Time spent immobile in the tonic immobility test; HeadNO: Number of scans when the quail passed its head through the wire of the front of the cage in the novel object test; Mount: Number of mounts in the sexual motivation test; AgrP: Number of aggressive pecks in the aggressive behavior test; GentleP: Number of gentle pecks in the aggressive behavior test; STAND: Time spent standing in the cage (general activity); W17: weight at 17 days; W65: weight at 65 days; AFEgg: Age of laying onset; NEgg: Number of eggs laid; WEgg: Mean egg weight. See Materials and Methods, Testing procedure for more details.

Spearman rank correlations (rho) between the different behavioral and production traits in F2 birds are presented in Table 3. A total of 18 correlations were significant (p< 0.05) at the phenotypic level. For behavioral traits, low to moderate correlations (-0.11 to 0.10) were observed between DistSR and DistIso (0.08) or AgrP (0.10), GentleP and AgrP (-0.11), HeadNO and Mount (0.10), and between TI and STAND (-0.07) or AgrP (-0.09). Phenotypic correlations were also observed between behavioral and production traits for DistSR and W17 (-0.10) or W65 (-0.09), TI and W17 (0.16) or W65 (0.12), GentleP and W17 (0.09), HeadNO and W65 (-0.09), and between W65 and STAND (-0.07). More marked phenotypic correlations were found between Mount and AgrP (0.51) and for production traits between W17 and W65 (0.54), W17 and WEgg (0.54), W65 and WEgg (0.43), AFEgg and NEgg (-0.56), and at a lesser extent W65 and NEgg (-0.10).

**Table 3:** Heritability values (diagonal in bold, h^2^ ± SE), genetic (above the diagonal, r_g_ ± SE) and phenotypic (r_p_) (below the diagonal) correlations between the various behavioral and production traits.

	DistSR	DistIso	TI	HeadNO	Mount	AgrP	GentleP	STAND	W17	W65	AFEgg	NEgg	WEgg
DistSR	0.19 ± 0.09*	0.16 ± 0.31	-0.35 ± 0.32	0.37 ± 0.20	0.32 ± 0.30	0.05 ± 0.33	0.29 ± 0.35	-0.23 ± 0.40	0.14 ± 0.60	0.03 ± 0.44	-0.71 ± 0.28*	0.32 ± 0.29	0.21 ± 0.57
DistIso	0.08*	0.34 ± 0.12*	-0.33 ± 0.28	-0.08 ± 0.28	-0.00 ± 0.28	0.35 ± 0.28	0.45 ± 0.28	-0.33 ± 0.31	-0.33 ± 0.44	-0.44 ± 0.34	-0.21 ± 0.43	0.04 ± 0.27	-0.22 ± 0.41
TI	-0.002	-0.05	0.21 ± 0.08*	0.24 ± 0.30	-0.22 ± 0.30	0.01 ± 0.32	-0.24 ± 0.34	-0.45 ± 0.31	0.76 ± 0.28*	0.79 ± 0.24*	0.74 ± 0.28*	-0.35 ± 0.28	0.38 ± 0.56
HeadNO	0.002	-0.01	0.06	0.36 ± 0.13*	0.89 ± 0.15*	0.63 ± 0.25*	0.63 ± 0.29*	-0.09 ± 0.33	0.49 ± 0.44	0.44 ± 0.28	-0.55 ± 0.40	0.61 ± 0.25*	0.48 ± 0.34
Mount	0.04	-0.008	-0.07	0.10*	0.49 ± 0.14*	0.90 ± 0.14*	0.73 ± 0.26*	-0.20 ± 0.31	0.18 ± 0.43	0.36 ± 0.28	-0.68 ± 0.32*	0.82 ± 0.14*	0.28 ± 0.33
AgrP	0.10*	0.05	-0.09*	-0.01	0.51*	***0.42 ± 0.15***	0.20 ± 0.42	-0.35 ± 0.33	-0.29 ± 0.42	0.00 ± 0.33	-0.38 ± 0.41	0.58 ± 0.22*	-0.11 ± 0.38
GentleP	-0.001	0.05	-0.01	0.03	0.05	-0.11*	0.39 ± 0.15*	-0.20 ± 0.31	0.45 ± 0.36	0.28 ± 0.33	-0.81 ± 0.24*	0.72 ± 0.24*	0.33 ± 0.31
STAND	-0.06	-0.05	-0.07*	0.01	-0.02	0.02	-0.03	0.13 ± 0.06*	-0.37 ± 0.37	-0.59 ± 0.33	0.05 ± 0.34	0.19 ± 0.31	-0.56 ± 0.45
W17	-0.10*	-0.04	0.16*	0.01	-0.02	-0.08	0.09*	0.007	0.48 ± 0.23*	0.72 ± 0.24*	0.48 ± 0.48	-0.06 ± 0.38	0.65 ± 0.34
W65	-0.09*	0.003	0.12*	-0.09*	-0.03	-0.03	0.05	-0.07*	0.54*	0.49 ± 0.20*	0.11 ± 0.49	-0.21 ± 0.42	0.48 ± 0.42
AFEgg	0.02	0.01	0.06	0.07	NC	NC	NC	-0.003	0.01	0.09	0.30 ± 0.15*	-0.88 ± 0.15*	0.46 ± 0.46
NEgg	-0.01	-0.02	-0.05	0.02	NC	NC	NC	0.04	-0.06	-0.10*	-0.56*	0.39 ± 0.12*	-0.17 ± 0.50
WEgg	-0.05	-0.02	0.05	0.10	NC	NC	NC	-0.005	0.54*	0.43*	-0.03	0.03	0.45 ± 0.21*

Significant parameters are shown with * (p< 0.05). DistSR: Distance travelled on the treadmill in the social reinstatement behavior test; DistIso: ​Distance travelled in periphery in the social isolation test; TI: Time spent immobile in the tonic immobility test; HeadNO:Number of scans when the quail passed its head through the wire of the front of the cage in the novel object test; Mount: Number of mounts in the sexual motivation test; AgrP: Number of aggressive pecks in the aggressive behavior test; GentleP: Number of gentle pecks in the aggressive behavior test; STAND: Time spent standing in the cage (general activity); W17: weight at 17 days; W65: weight at 65 days; AFEgg: Age of laying onset; NEgg: Number of eggs laid; WEgg: Mean egg weight. NC: Non-computable

### Genetic parameters

Except for STAND (h^2^ = 0.13), the estimated heritability of behavioral traits was moderate to high (Table 3). It ranged from 0.19 to 0.36 for DistSR, DistIso, TI and HeadNO and from 0.39 to 0.49 for Mount, AgrP and GentleP. For production traits, the heritability level was moderate for AFEgg (0.30), but high ranging from 0.39 to 0.49 for W17, W65, NEgg, and WEgg. 

Some of the most significant correlations observed at the phenotypic level were confirmed at the genetic level at a higher value. For instance, Mount and AgrP had a strong positive genetic correlation (0.90). This was also the case for TI and W17 (0.76) as well as W65 (0.79) and W17 and W65 (0.72). As expected, AFEgg and NEgg had a strong negative genetic correlation (-0.88). Additional significant correlations were found at the genetic level while they were not found at the phenotypic level. HeadNO was positively correlated with AgrP (0.63) and GentleP (0.63). Mount was positively correlated with HeadNO (0.89) and GentleP (0.73). On the other hand, AgrP and GentleP were not genetically correlated. In addition, other significant genetic correlations were found between some production and behavioral traits. In particular, AFEgg was negatively correlated with Mount (-0.68), GentleP (-0.81) and DistSR (-0.71) and positively correlated with TI (0.74), whereas NEgg was positively genetically correlated with Mount (0.82), GentleP (0.72) and also with AgrP (0.58) and HeadNO (0.61). 

## Discussion

With the exception of sexual behavior and aggressiveness which were highly correlated at the phenotypic level, phenotypic correlations between behavioral traits were low. This underlines the necessity of a multi-criteria approach in order to analyze and interpret the behavior of animals correctly, using a series of tests to measure different aspects of their responses to a given situation. In addition, our results show a partly common genetic regulation of several behavioral traits including aggression, sexual motivation and response to an unknown object. Several behavioral traits (socio-sexual and emotional reactivity traits) also exhibited significant genetic correlations with production traits (body weight and egg production) showing that selection on production traits may affect behavior and vice versa.

The estimated heritability values obtained in this study show the significant contribution of genetics to the phenotypic variability of all the behavioral traits. Indeed, heritability was moderate (0.20<h^2^<0.40) to high (h^2^>0.40) with the exception of the time spent standing in the cage which had a low heritability (0.13). These values were also close to those obtained with production traits, ranging from 0.30 to 0.49. Our estimates are in part consistent with previous studies even if it is known that several factors such as age of the bird, environmental conditions (e.g. housing in group or individually) or testing procedure affect the heritability coefficients. When analyzing, the first eight generations of the divergent selection for the distance run on the treadmill to rejoin conspecifics without log-transformation, Mills and Faure [9] showed that the heritability of this trait ranged from 0.16 to 0.38. Furthermore, in the same study, they showed that after the first eight generations of divergent selection for the duration of tonic immobility, this trait’s heritability ranged from 0.09 to 0.23. Our estimates of 0.19 and 0.21 for the distance run on the treadmill and the duration of tonic immobility, respectively, were therefore consistent. Our estimated heritability for the response to social isolation (0.34) was similar to those observed by Faure [32] and Agnvall et al. [33] in chickens tested in an open field (0.39 and 0.32, respectively), and between the values observed by Rodenburg et al. [34] in hens: 0.49 at 5 weeks of age, but 0.15 at 29 weeks of age. The heritability obtained in the present study for sexual behavior, estimated by the number of mounts (0.49), was lower than that obtained by Nol et al. [35] (0.64), but within the upper limit of other previous estimations. Siegel [36] found a heritability value of 0.34 in divergent lines of chickens selected on their mating frequency, while Gerken and Petersen [37] observed realized heritability values ranging from 0.05 to 0.44 in Japanese quail. Regarding pecking behavior, namely gentle and aggressive pecking, heritability values in the present study (0.39 and 0.42, respectively) were much higher than those reported by Rodenburg et al. [34] in laying hens (0.01-0.02 for aggressive pecking and 0.08-0.16 for gentle pecking according the age of the bird). Our estimation is also higher than the heritability found by Nol et al. [35] for fighting in Japanese quail (0.31). The higher heritability values in the present study for sexual and aggressive behaviors may be a consequence of using a lure or a mirror during the tests instead of live individuals, leading to a more homogeneous response between tested individuals and reducing the variability linked to interactions between individuals. It could also be a consequence of the housing in isolation from 23 days of age, reducing the influence of social interactions initiated before testing on the response obtained during the tests. Furthermore, in the case of aggressive behavior, the motivation to peck toward a mirror, i.e., toward the reflection of the tested male, is probably different from the motivation to peck a conspecific in layers. As expected, growth and laying traits had moderate to high levels of heritability (0.30-0.49) as already reported by Nestor et al. [38], Marks [39] and Silva et al. [40] for body weight and Gerken and Petersen [37] for the age of laying onset.

Several phenotypic correlations between behavioral traits were observed. Most of these were weak (between -0.11 and 0.10), highlighting that each test and variable measured a different aspect of quail behavior. Thus, we observed a low phenotypic correlation and no genetic correlation between the distances run to rejoin conspecifics in the test measuring social reinstatement behavior or in the periphery in the social isolation test. This would suggest these two tests measure different components of social motivation, depending on whether the animals are in visual and auditory contact with conspecifics or not. Furthermore, the absence of correlation between the duration of tonic immobility and response to a novel object is in line with previous studies performed in Japanese quail [22,41]. This suggests that these two tests measure different components of emotional reactivity, probably associated with different cognitive evaluation of the situation [10,42]. The responses of birds are likely to be influenced by their ability or inability to control the stressful situation during the test. During the tonic immobility test, individuals are restrained while during the novel object test they can express a larger range of behavioral responses. Our results also show that although the selection criteria (i.e. the distance travelled on the treadmill) involved a high level of locomotor activity, it does not seem to be related to the quail’s general activity. Indeed, we observed low phenotypic and genetic correlations between the distance travelled during the tests assessing social motivation and the time spent standing in cages. 

High phenotypic and genetic correlations were observed between sexual motivation (Mount) and aggressive pecking (AgrP). These correlations support the relationships already described in Japanese quail and domestic fowl. Indeed, aggressive and sexual behaviors show similar patterns in males [43] and both behaviors are largely regulated by testosterone [28,44]. Moreover, quail lines differing in their mating ability also differ in their ability to be dominant, both behaviors being positively associated [45]. The response to a novel object (HeadON) also had a high positive genetic correlation with aggressiveness (AgrP) and, to a lesser extent, with sexual motivation (Mount). When testing the reaction to a novel object, we measured the balance between avoidance (fear) and attraction (exploration) or even aggressiveness since the unknown object placed in front of the cage could be considered as a threat by quail thus resulting in an aggressive reaction rather than avoidance in fearless animals. Moreover, the use of a mirror and a lure rather than live birds in the aggressive and sexual behavior tests could have enhanced the novelty effect due to the testing situation and could explain why the responses in these two tests were so strongly correlated to the novel object test. Only weak phenotypic correlations and no genetic correlation were found between the number of gentle pecks (GentleP) and aggressive pecks (AgrP). This result suggests that the two traits are not the low and high levels of expression of a same phenotype, but rather behaviors with different motivations: positive interactions (search of contact) in the first case and agonistic interactions in the second. The positive genetic correlation between the number of gentle pecks and the number of scans in which quail passed their heads through the wire of the front of the cage could be a sign of looking for contact with the unknown object (i.e. attraction) in birds with a low level of fear and aggressive behavior.

Interestingly, several correlations between behavioral and production traits were highlighted in this study. Positive phenotypic and genetic correlations were found between emotional reactivity estimated by the duration of tonic immobility and weight at 17 and 65 days. Previous studies on phenotypic links between tonic immobility duration and growth have shown contradictory results: an absence of correlation between these traits [46,47] or a negative correlation [48] in Japanese quail. However, a study in a F2 cross in laying hens found a positive phenotypic relationship between these two traits [49]. Moreover the high positive genetic correlation we observed between tonic immobility duration and weight strongly suggests that common genes control growth and tonic immobility duration. This common genetic basis has also been suggested by a study in laying hens showing a co-localization of QTLs linked to growth or fear responses [50] and a possible pleiotropic effect of a limited region of chromosome 1 affecting both [12]. Our result implies that birds with higher TI might react to frightening situations by behavioral inhibition making them less active and consequently heavier. If this is the case, the relationship would only be observed in frightening situations since there was no significant genetic correlation between time spent standing (STAND) and tonic immobility duration. Other mechanisms including differences in metabolism or locomotor activity could be involved in this positive correlation we observed between tonic immobility and weight. Emotional reactivity seems to affect another production trait since we observed a strong positive genetic correlation between tonic immobility duration and the age of laying onset which implies that the selection of birds with high emotional reactivity could lead to delayed maturity. A positive genetic correlation was also found between the number of scans when the quail passed its head through the wire of the front of the cage to face the novel object and the number of eggs laid. This result implies that birds selected for a low level of emotional reactivity could show more abundant egg laying. The selection criteria of our divergent lines, i.e. the distance run on a treadmill to rejoin congeners, showed a strong negative correlation with the age of laying onset, which means that birds with high social motivation lay earlier. Such a relationship has already been observed in Japanese quail by Marin et al. [51]. In that study, social motivation was assessed by the time taken by individuals to join conspecifics in a T-shaped maze with two exits, one empty, the other one containing conspecifics. The most socially motivated individuals showed earlier puberty, i.e. a lower age at onset of egg laying, and laid more eggs per day. In our study, the number of mounts and of gentle pecks were positively correlated with the age of onset of laying and the number of eggs, but this latter was also positively correlated with aggressive pecking to a lesser extent. These genetic results suggest that the sexual activity and reproductive capacity of the birds would be related directly or indirectly to their social motivation. This is in line with previous observations by Burns et al. [21] showing that social motivation is related to an increase in reproductive capacity. 

Large differences were observed between the behaviors of the HSR and LSR divergent lines in the social reinstatement test, isolation test, but also in the emotional reactivity test. This last result for tonic immobility differs from most previous results e.g.[52], although a similar result was obtained by Schweitzer and Arnould [22]. Indeed, a constraint on this trait was applied in the selection procedure to reduce the risk of co-selection for social reinstatement behavior and duration of tonic immobility [9]. In the same time, tonic immobility values were quite low, showing a very low level of fear in these birds. Given the significant behavioral differences between the HSR and LSR quail, the very low phenotypic and genetic correlations between the corresponding behaviors in the F2 population were to some extent not expected. Between line differences result both from direct and indirect effects of selection, but also from random effects accumulated during selection such as genetic drift. Crossing animals results in genetic recombination so that random associations are no longer present. Our results underline that the balance between attraction (exploration) and fear (avoidance) and therefore the bird’s response when faced with a specific situation may be considerably different between extremely divergent birds and an F2 population sharing a more homogenous genetic background.

## Conclusion

This study brings new insights to the complex determinism of social and fear behaviors in birds and their relationships with production traits. The genetic correlations found thus suggest two groups of traits: one involving socio-sexual behaviors (social reinstatement behaviors, mounts, aggression) that would be linked to high reproductive abilities (early onset of laying and high number of eggs) and a second consisting of tonic immobility duration related to emotional reactivity that is linked to high body weight but delayed onset of laying. Although these results need to be confirmed in other commercial crossbreds and species of interest, they highlight that the joint genetic analyses of behavior and production traits is a key step in establishing breeding programs. Finally, with the exception of the correlation between sexual motivation and aggressiveness or reaction to a novel object and between tonic immobility and weights, the correlations significant at the genetic level were not found at the phenotypic level which suggests a strong impact of environmental factors on the behaviors studied. These environmental factors could include epigenetic effects. This highlights that both the genetics of birds and their breeding conditions must be controlled to enhance welfare and animal production, being aware that factors such as the age of the birds or housing conditions (in group of single sex, mixed sex or individually) influence greatly social and emotional behavior, but also production performances.

## Supporting Information

Figure S1 **Histograms of distribution for the behavioral and production traits in F2 population.** Histogram of distribution for (**A**) DistSR the distance travelled on the treadmill (in arbitrary units) in the social reinstatement behavior test, (**B**) DistIso the distance travelled in periphery (in cm) in the social isolation test, (**C**) TI the time spent immobile (in s) in the tonic immobility test, (**D**) HeadNO the number of scans when the quail passed its head through the wire of the front of the cage in the novel object test, (**E**) Mount the number of mounts in the sexual motivation test, (**F**) AgrP the number of aggressive pecks in the aggressive behavior test, (**G**) GentleP the number of gentle pecks in the aggressive behavior test, (**H**) STAND the time spent standing in the cage (in %) during the general activity test, (**I**) W17 the weight (in g) at 17 days, (**J**) W65 the weight (in g) at 65 days, (**K**) AFEgg the age of laying onset (in days), (**L**) NEgg the number of eggs laid, (**M**) WEgg the mean egg weight (in g).
(DOCX)
