# Supplementary figures and images for: Evidence of Phenotypic and Genetic Relationships between Sociality, Emotional Reactivity and Production Traits in Japanese Quail
Source: PLoS One. 2013 Dec 4;8(12):e82157. doi: 10.1371/journal.pone.0082157 (PMC3852745; doi:10.1371/journal.pone.0082157)

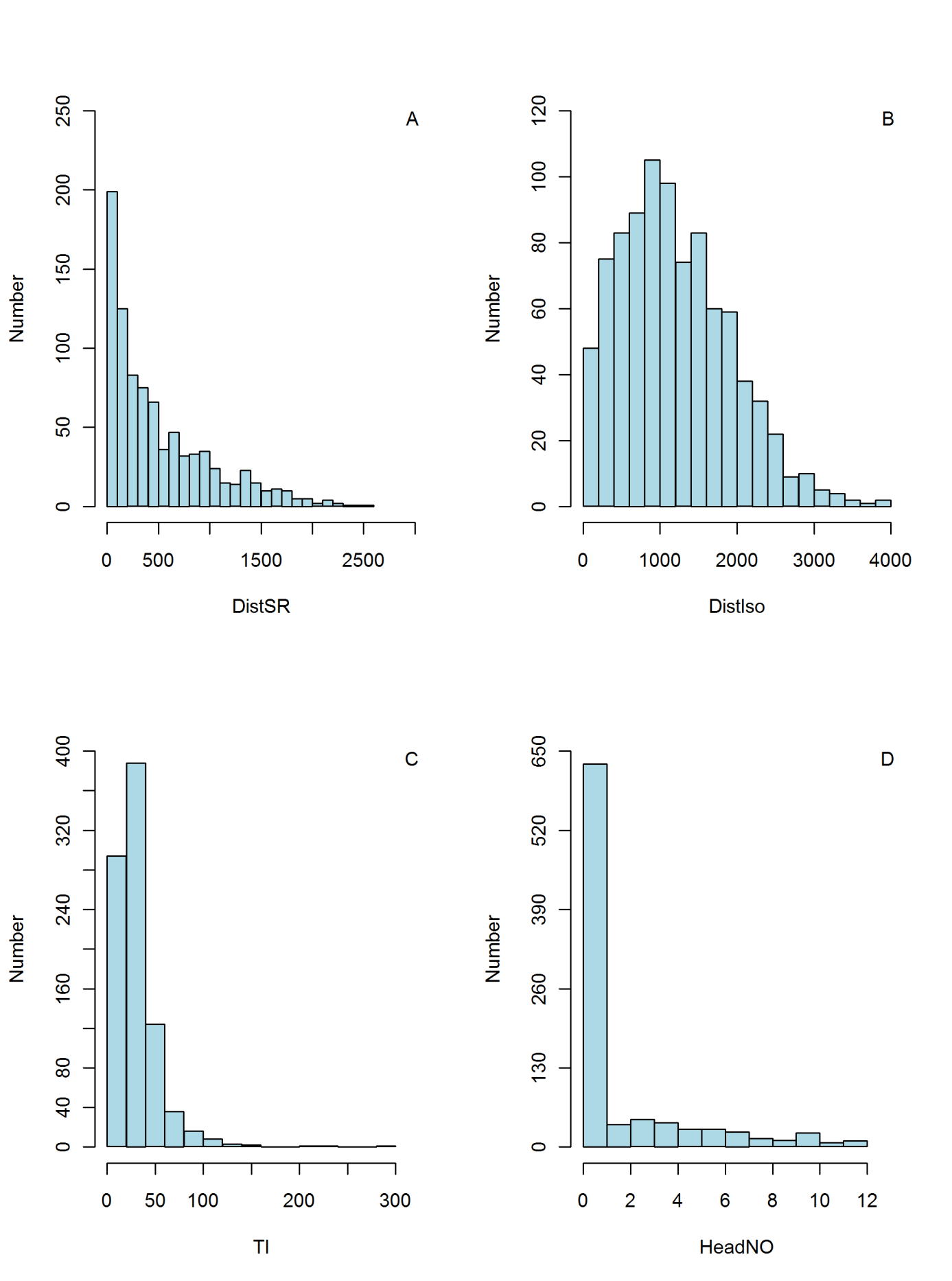


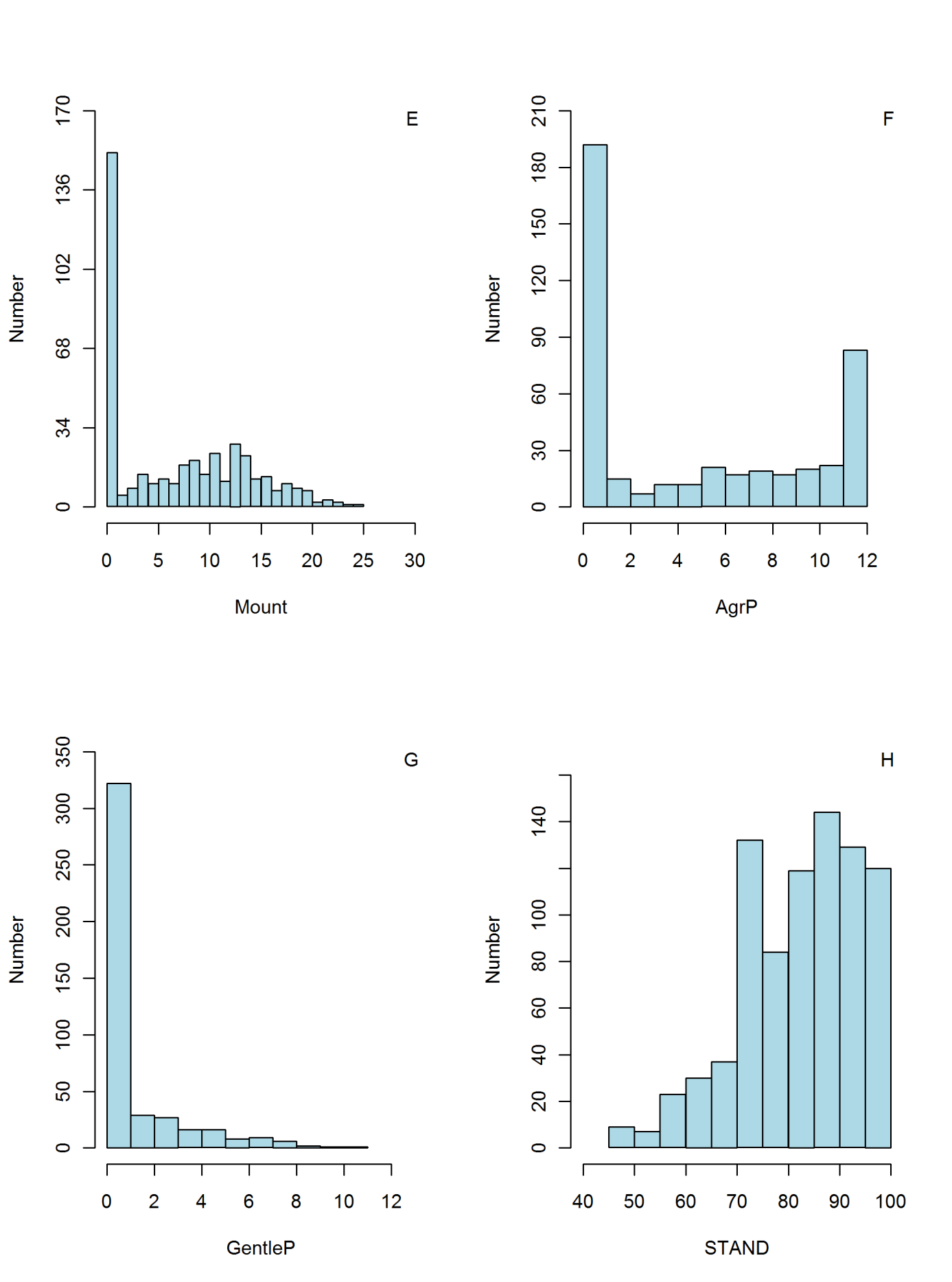


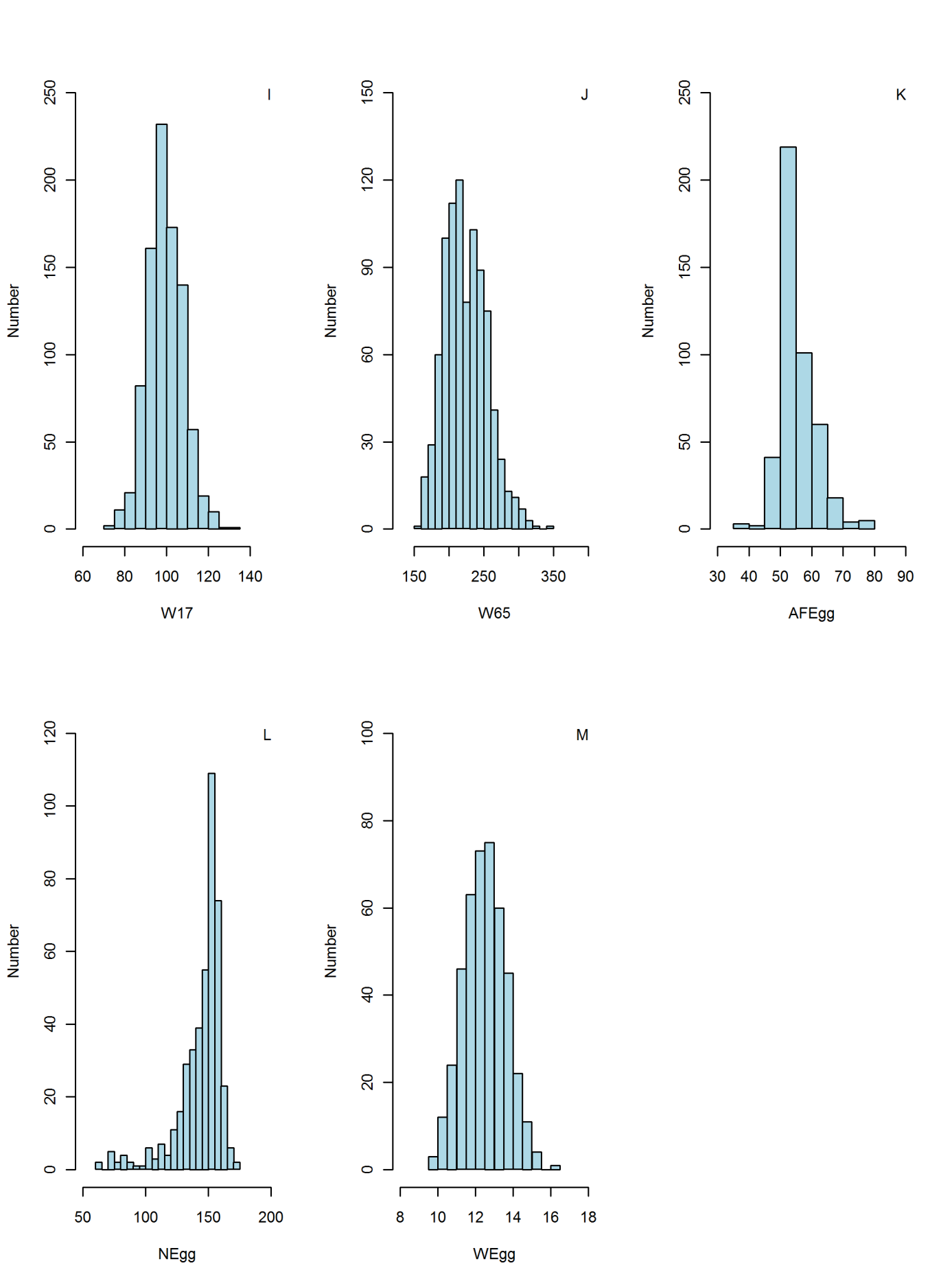

Supplement: Figure S1 — Histograms of distribution for the behavioral and production traits in F2 population. Histogram of distribution for (A) DistSR the distance travelled on the treadmill (in arbitrary units) in the social reinstatement behavior test, (B) DistIso the distance travelled in periphery (in cm) in the social isolation test, (C) TI the time spent immobile (in s) in the tonic immobility test, (D) HeadNO the number of scans when the quail passed its head through the wire of the front of the cage in the novel object test, (E) Mount the number of mounts in the sexual motivation test, (F) AgrP the number of aggressive pecks in the aggressive behavior test, (G) GentleP the number of gentle pecks in the aggressive behavior test, (H) STAND the time spent standing in the cage (in %) during the general activity test, (I) W17 the weight (in g) at 17 days, (J) W65 the weight (in g) at 65 days, (K) AFEgg the age of laying onset (in days), (L) NEgg the number of eggs laid, (M) WEgg the mean egg weight (in g). (DOCX) [file pone.0082157.s001.docx]
